# Supplementary material for: Controllable two-dimensional movement and redistribution of lithium ions in metal oxides
Source: Nat Commun. 2019 Jun 28;10:2888. doi: 10.1038/s41467-019-10875-w (PMC6599050; doi:10.1038/s41467-019-10875-w)
Supplement: Supplementary file 1 — Supplementary Information [file 41467_2019_10875_MOESM1_ESM.pdf]

Supplementary Information to:

Controllable two-dimensional movement and redistribution of lithium ions in metal oxides

Tang, X. F. et al.

Supplementary Information

Supplementary Figures

Supplementary Fig.1 Characterization of the original WO_3 film.

Supplementary Fig.2 SEM micrographs of a selected area of the WO_3 film during the entire process.

Supplementary Fig.3 In-situ observation of the WO_3 film transmittance on an ITO glass substrate.

Supplementary Fig.4 In-situ step profile characterization of the WO_3 film on an ITO glass substrate by AFM.

Supplementary Fig.5 I-V curves of a pure ITO glass substrate and the same ITO glass coated with the Z-Moved WO_3 film measured by Keithley 2400 Source Meter. **Supplementary Fig.6** Control of the 2D horizontal movement of Li ions in the WO_3 film by another current flowing pattern “Main road and its branches”.

Supplementary Fig.7 In-situ observation by AFM of the diffusion process of Li ions in the WO_3 film with uneven thickness.

Supplementary Fig.8 A curtain-like smart window based on the 2D movement of Li ions in the WO_3 film.

Supplementary Tables

Supplementary Table 1 To date, successful attempts of the controllable 2D movement of Li ions in metal oxides according to the ‘current-driving model’.

Supplementary Table 2 Besides Li ions discussed in the manuscript, 2D movement of four more cations in WO_3 films were also achieved according to the ‘current-driving model’, namely H^+, Na^+, Zn^{2+} and Ca^{2+}.

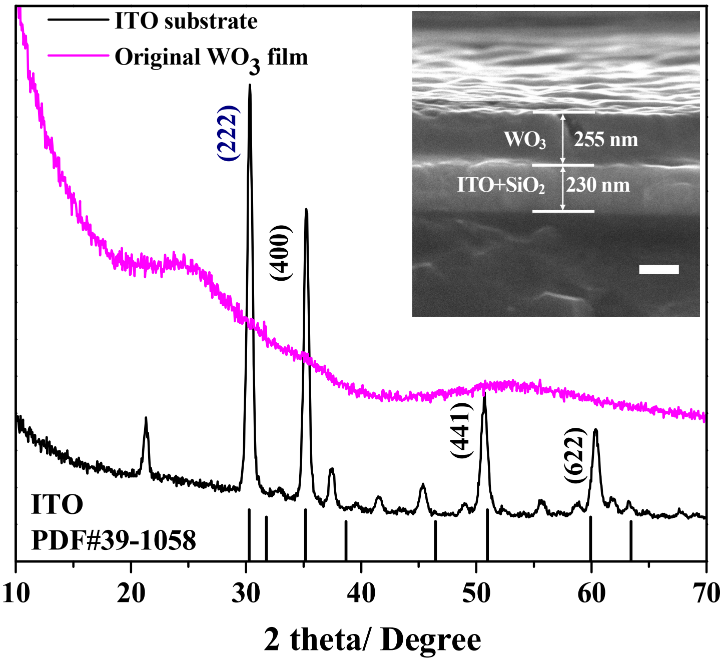

Supplementary Fig.1 Characterization of the original WO_3 film. XRD spectra of the WO_3 film deposited on an ITO glass substrate, showed that the original WO_3 film was amorphous. The inset was a cross-sectional SEM image of the sample, suggesting the thicknesses of the WO_3 film and the bottom ITO conducting layer (excluding a thin SiO_2 film as the barrier layer, approximately 30 nm given in the product report) were 255 nm and 200 nm respectively, and their interfacial contact was good. Scale bar, 200 nm.

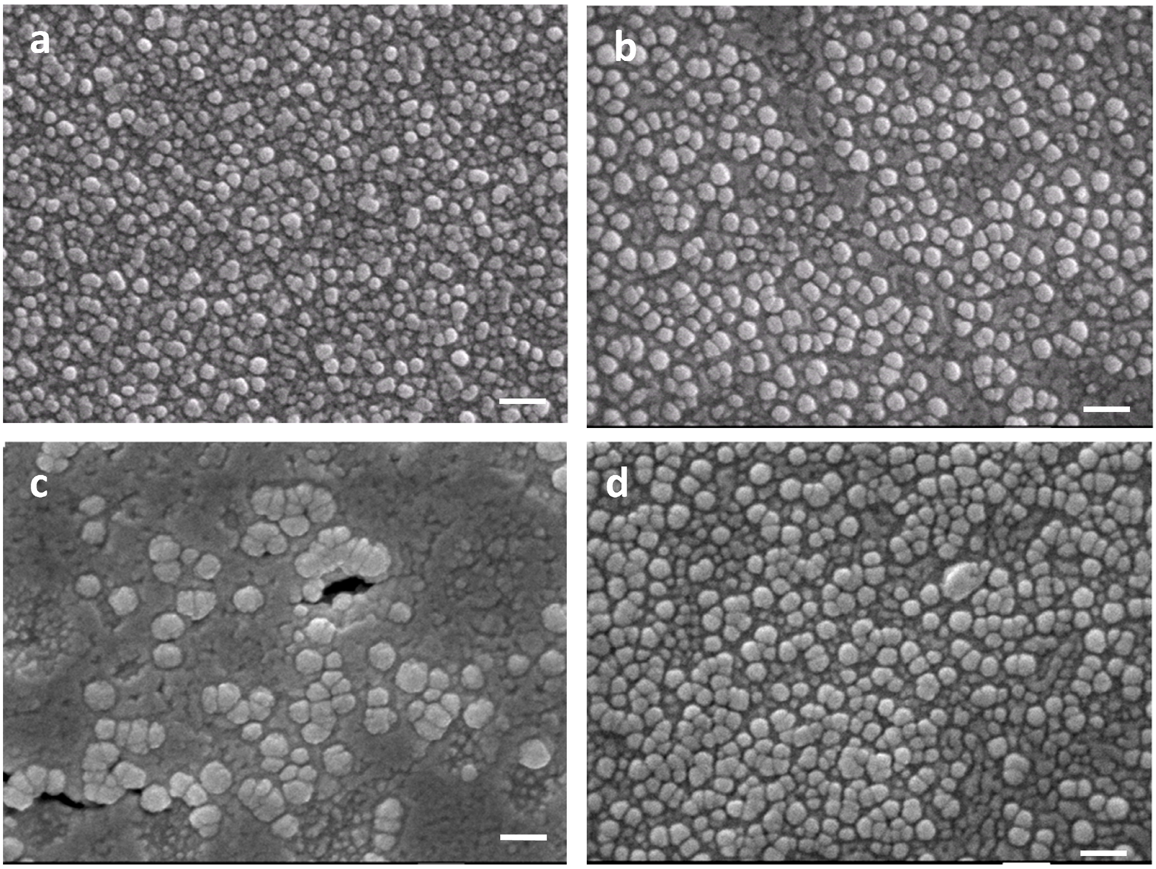

Supplementary Fig.2 SEM micrographs of a selected area of the WO_3 film during the entire process. **a.** Original state, **b.** Z-Moved state, **c.** R-L Moved state, **d.** the self-diffusion state. Scale bars, 100 nm. The original film was composed of uniformly-distributed fine particles. Particles in Z-moved film became larger and some area was stretched flat. To R-L Moved state, the film surface got flat with scattered big particles, their agglomerates and fissures, confirming that much more Li ions were gathering in this area. Film morphology of the 20 min self-diffussion state got similar back with Z-moved state (b).

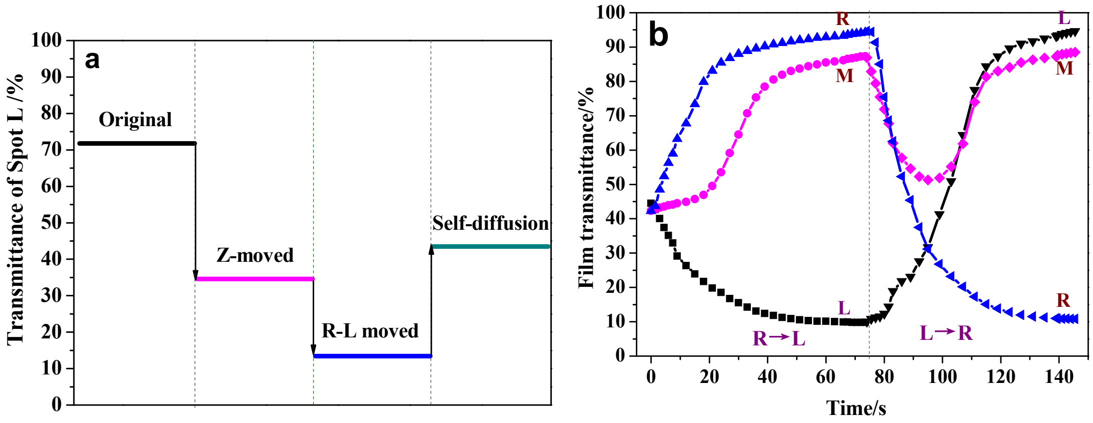

Supplementary Fig.3 In-situ observation of the WO_3 film transmittance on an ITO glass substrate. **a.** Transmittance variation of Spot L (signed in manuscript Fig.2b) throughout the entire process. **b.** Film transmittance evolution of Spot L, M and R (signed in manuscript Fig.2b) during the first two cycles of Li ions moving from the right to the left (**R-L**) and reversely from the left to the right (**L-R**). The transmittance variation clearly told the amount and the moving path of Li ions that were involved in the entire process. In addition, the film transmittance increased by 8.9% comparing Z-Moved state with the 20 min self-diffusion state during Li ion one-cycle horizontal movement, indicating that dynamic exchanges of Li ions at the solid-liquid interface did happen, resulting into the obvious self-bleaching phenomenon. Furthermore, during the second cycle (L-R), transmittance of Spot M first decreased and then increased during the migration process, which was different from the first cycle (R-L, showing a monotonic increase), suggesting that the migration paths of Li ions in the WO_3 film during the first two cycles were different.

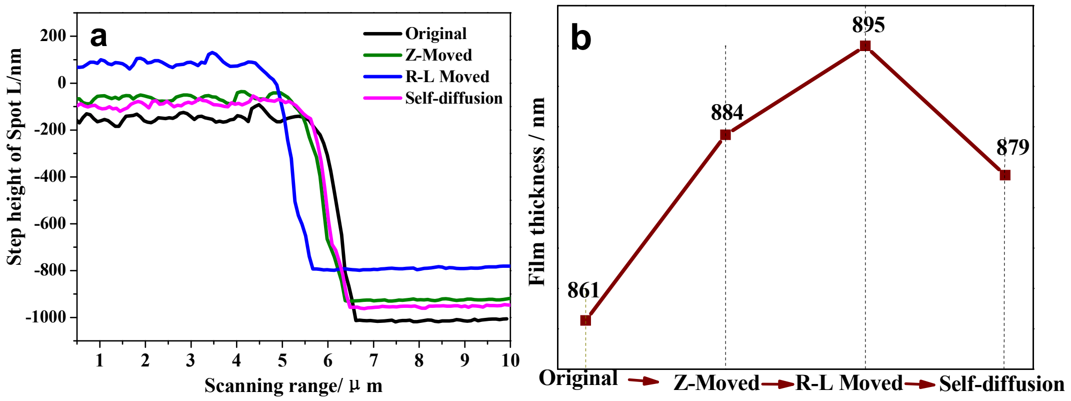

Supplementary Fig.4 In-situ step profile characterization of the WO_3 film on an ITO glass substrate by AFM. **a.** In-situ observation of the step profile of the WO_3 film at the left-most end throughout the entire process. In P2, Li ions moved from the right to the left and accumulated at the left end of the film. **b.** The film thickness calculated from the step profile. Comparing with the step profile of the original WO_3 film, both sides of the step in Z-Moved state were lifted, due to the volume expansion of the vertical injection of Li ions. Overall, the film thickness in Z-Moved state (884 nm) was increased by 23 nm from 861 nm in Original state. When Li ions accumulated at the left end after the 2D movement, the film thickness was further increased by 11 nm. At this moment, both sides of the step were further greatly lifted, suggesting much more Li ions were accumulating at the left-most end than Z-Moved state. Finally, the step height turned back to 879 nm at the 20 min self-diffusion state.

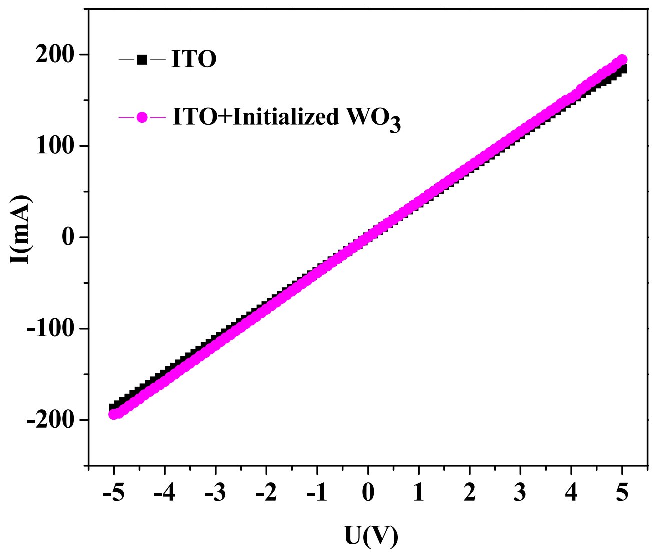

Supplementary Fig.5 I-V curves of a pure ITO glass substrate and the same ITO glass coated with the Z-Moved WO_3 film measured by Keithley 2400 Source Meter. Excluding all random errors generated during the film fabrication process and the testing process, the two curves could be thought to approximately coincide, which confirmed that resistance of the Z-Moved WO_3 film was much higher than that of the ITO conducting layer. This was also verified that resistance of the Z-Moved WO_3 film was 0.4 MΩ, whereas that of the ITO conducting layer was 20 Ω, as measured by a digital multimeter.

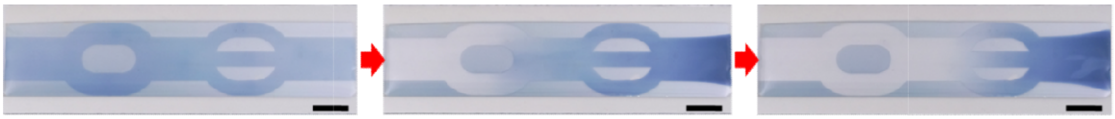

Supplementary Fig.6 Control of the 2D horizontal movement of Li ions in the WO_3 film by another current flowing pattern "Main road and its branches". Scale bars, 1cm.

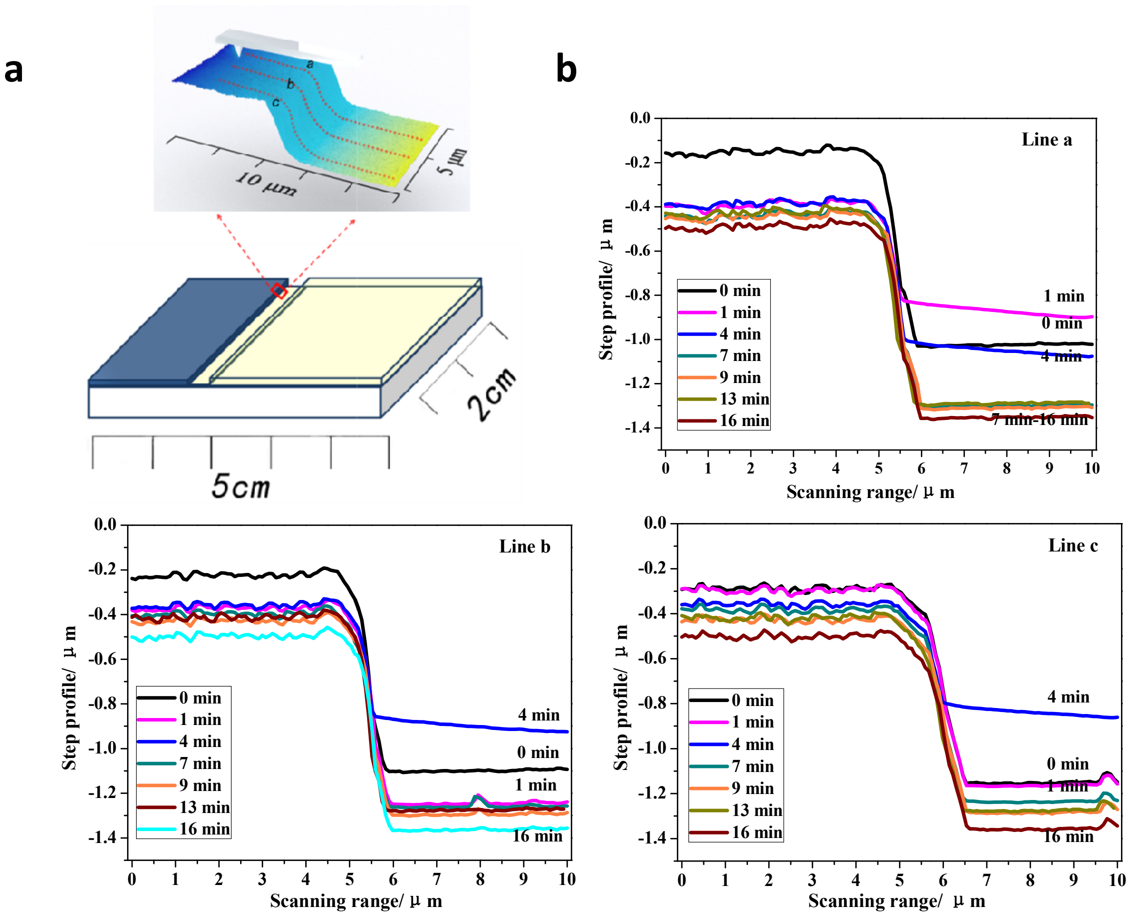

Supplementary Fig.7 In-situ observation by AFM of the diffusion process of Li ions in the WO_3 film with uneven thickness. A very large concentration difference was deliberately fabricated through the controllable 2D movement of Li ions in WO_3 by the 'current-driving model'**. a.** Schematic of the deliberately-designed uneven WO_3 film and the operando of the in-situ observation, where Li ions accumulated in the blue area by the 'current-driving model'. Between the blue area and the yellow area was a gully fabricated with mask method. Lines a, b and c denoted the selected sections whose profile evolutions were correspondingly shown in **b.** Evolution of the whole step profile during the diffusion process was compiled to be a video (Supplementary movie 3). It can be clearly seen that this diffusion process did not happen simultaneously, but first started at Line a, then to Line b and then to Line c. Notably, in this diffusion process, moving waves of Li ions similar to water waves were observed.

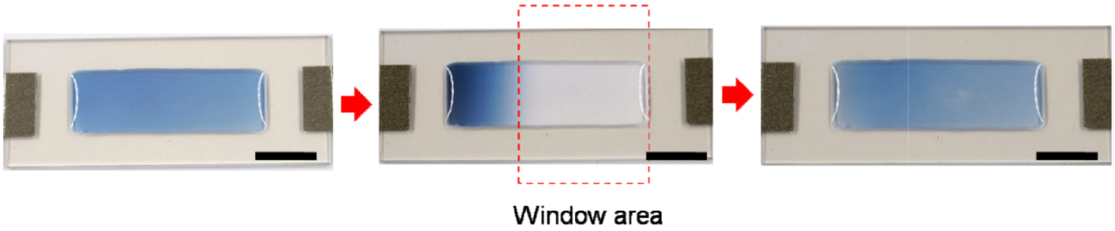

Supplementary Fig.8 A curtain-like smart window based on the 2D movement of Li ions in the WO_3 film. The modulation rate of this window can be controlled by the amount of the injected Li ions in the Z-Moved process and the window area is customizable by the ‘current-driving model’ like a curtain being pulled open.

Supplementary Table 1 To date, successful attempts of the controllable 2D movement of Li ions in metal oxides according to the 'current-driving model'.

For WO_3 and MoO_3, color changes are visible when Li ions move in the films. Optical photos of the R-L Moved state of the films were shown.

For TiO_2, color changes are not obvious, optical and SEM photos were both listed. **a.** Optical photos corresponded to Original, Z-Moved and R-L Moved states respectively from left to right. **b. c. d and e.** Corresponding SEM images of the dashed square box areas marked in **a**.

For Nb_2O_5@ graphite, color changes cannot be observed and SEM results were presented, **a.** Original state, **b.** Z-Moved state, **c.** SEM image from the left area (L) of R-L Moved state, **d.** SEM image from the right area (R) of R-L Moved state, **e.** Illustration of the film at R-L Moved state after Li ions were transported from right to left, where the two dashed boxes (signed as L and R) denoted the SEM testing areas. Scale bars in TiO_2 SEM images, 400 nm; scale bars in Nb_2O_5@ graphite SEM images, 1 μm.

Metal oxide	Electrolyte	Experimental results	Move or not
WO_3	Li^+ (1mol/L PC solution)	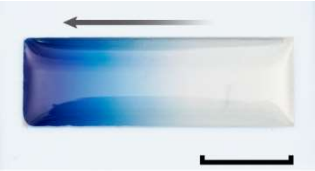	√
MoO_3	Li^+ (1mol/L PC solution)	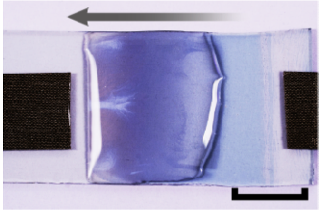	√
TiO_2	Li^+ (1mol/L PC solution)	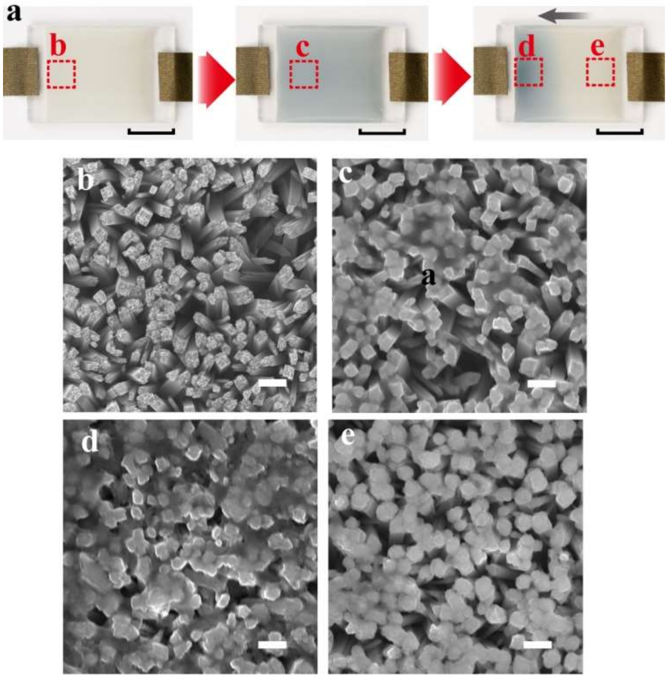	√

Nb_2O_5@ graphite	Li^+ (1mol/L PC solution)	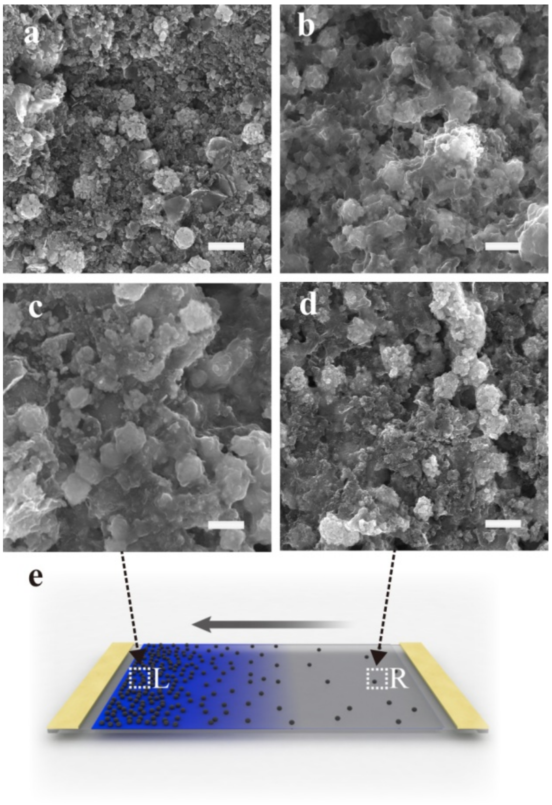	√

Supplementary Table 2 Besides Li ions discussed in the manuscript, 2D movement of four more cations in WO_3 films were also achieved according to the 'current-driving model', namely H^+, Na^+, Zn^{2+} and Ca^{2+} .

Metal oxide	Ion type	Proof	Move or not
**WO_3**	**H^+**(5% H_2O solution)	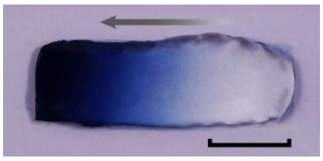	√
	**Na^+**(10% H_2O solution)	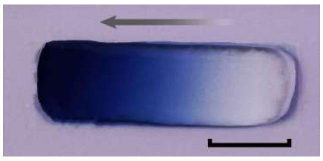	√
	**Zn^{2+}**(10% H_2O solution)	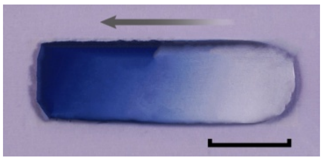	√
	**Ca^{2+}**(10% H_2O solution)	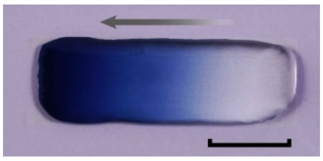	√

Supplementary Movies

Supplementary movie 1

Key elements exploration in the 'current-driving model' through the visible 2D movement of Li ions in WO_3 films.

Supplementary movie 2

Experimental process showing control of the 2D movement and the distribution of Li ions in WO_3 by the electrolyte distribution.

Supplementary movie 3

In-situ observation by AFM of the diffusion process of Li ions in the WO_3 film with uneven thickness. A very large concentration difference was deliberately fabricated through the controllable 2D movement of Li ions in WO_3 by the 'current-driving model'.
