# Supplementary material for: Controllable two-dimensional movement and redistribution of lithium ions in metal oxides
Source: Nat Commun. 2019 Jun 28;10:2888. doi: 10.1038/s41467-019-10875-w (PMC6599050; doi:10.1038/s41467-019-10875-w)
Supplement: Supplementary file 3 — Description of Additional Supplementary Files [file 41467_2019_10875_MOESM3_ESM.pdf]

Description of Additional Supplementary Files

Supplementary Movie 1 Key elements exploration in the 'current-driving model' through the visible 2D movement of Li ions in WO_3 films.

Supplementary Movie 2 Experimental process showing control of the 2D movement and the distribution of Li ions in WO_3 by the electrolyte distribution.

Supplementary Movie 3 In-situ observation by AFM of the diffusion process of Li ions in the WO_3 film with uneven thickness.
